# Thumbs up: Needle marker technique for preoperative localisation of radiolucent foreign bodies with ultrasonography^⋆^

**DOI:** 10.1016/j.ijscr.2020.06.020

**Published:** 2020-06-15

**Authors:** Valliappan Muthukumar, David Bodansky, Ravindra Badge

**Affiliations:** Warrington General Hospital, United Kingdom

**Keywords:** Foreign bodies, Radio- lucent, Ultrasonography, Needle, New technique, Case report

## Abstract

**Background:**

A 26-year-old man presented with pain and swelling after pricking his right thumb pulp with a thorn, gardening three-weeks previously.

**Technique:**

After a thumb digital ring block with local anesthetic, a linear array ultrasound (US) transducer (frequency XX) identified a foreign body (FB) in both longitudinal and transverse planes. This was a hyperechoic FB, 5mm in length with surrounding hypoechoic shadow, denoting granulation tissue and fluid collection. Two 20G needles were passed orthogonally, in-line with the US transducer, from the fingertip and ulnar aspect. The needle tips were placed touching, just deep to the FB, preventing displacement. A longitudinal incision was made over the intersection, revealing the FB.

**Discussion:**

Here, we describe peri-operative localization of radiolucent FBs with ultrasonography needle placement in superficial or deep planes as an adjunct to necessary surgical exploration. This method has a small learning curve, reduces the required incision and helps prevent pushing the FB deeper during dissection.

## Highlights

• Using Ultrasonography for identification of wooden or glass foreign body- case report.
• Easy localisation of foreign body with needles to exactly place incision and precise procedure.
• Can be used in any part of appendicular skeleton.

## 1 Background

A 26-year-old man presented with pain and swelling after pricking his right thumb pulp with a thorn, gardening three-weeks previously. The patient requested intervention due to pain and impact on his manual engineering work. Hand radiographs were normal, with no identifiable radio-opaque material.

This work has been reported in line with the SCARE criteria [6].

## 2 Technique

**Fig. 1:**
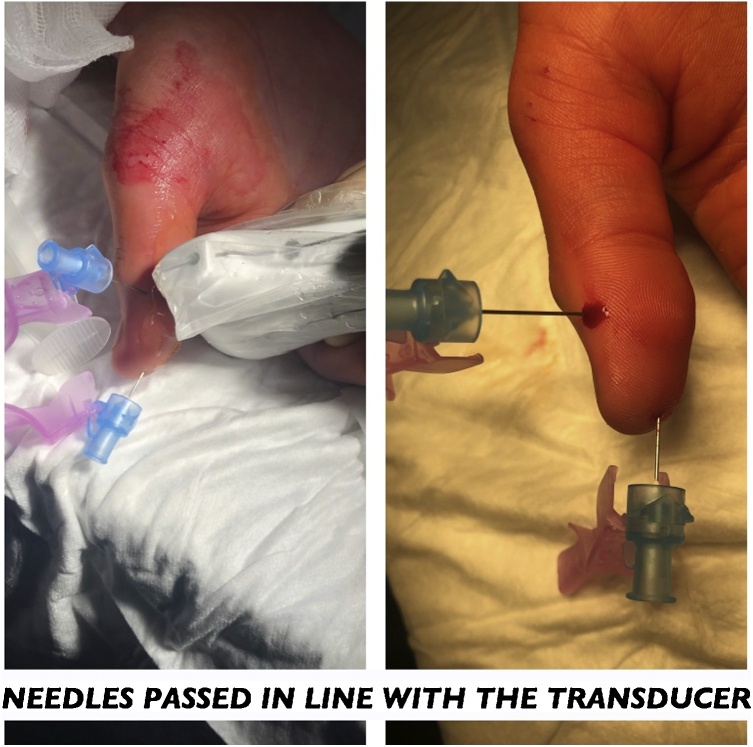
Insertion of orthogonal needles under ultrasound guidance, with needle tips just deep to the FB.

**Fig. 2:**
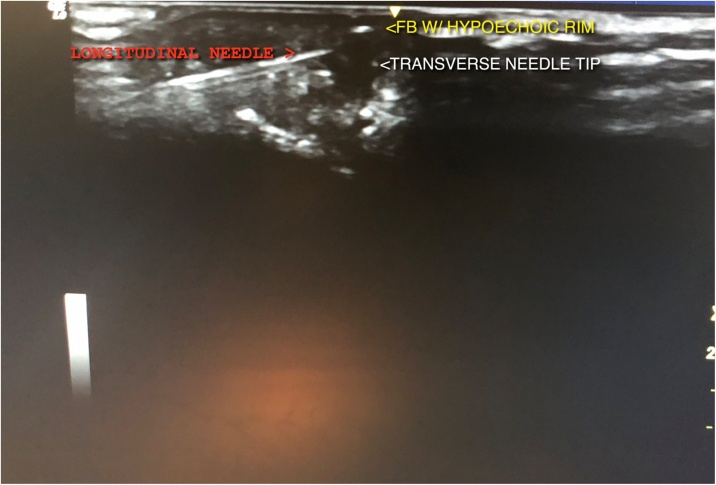
Identification of a hyperechoic foreign body on ultrasound with surrounding granulation tissue. Needle tips placed deep to foreign body, preventing displacement. Tips of the transverse and longitudinal needles can be appraciated.

After a thumb digital ring block with local anaesthetic, a linear array ultrasound (US) transducer (frequency XX) identified a foreign body (FB) in both longitudinal and transverse planes. This was a hyperechoic FB, 5 mm in length with surrounding hypoechoic shadow, denoting granulation tissue and fluid collection (Fig. 2). Two 20 G needles were passed orthogonally, in-line with the US transducer, from the fingertip and ulnar aspect (Fig.1). The needletips were placed touching, just deep to the FB, preventing displacement (Fig. 2). A longitudinal incision was made over the intersection, revealing the FB.

## 3 Discussion

One-third of FBs are missed during initial examination [1]. Wood, glass and metal account for 95 % of retained FBs. Radiographs identify 95 % of metal and glass but 15 % of wood [1]. Retained FBs can injure neurovascular bundles [2]. Exploration for radiolucent FBs can be challenging. Ultrasonography can help detect radiolucent FBs and previously has been used in real time during aseptic procedures [3,4]. Hitherto, single needle markers have been placed with XR in feet for radio-opaque FBs pre-surgically [5].

Here, we describe peri-operative localization of radiolucent FBs with ultrasonography needle placement in superficial or deep planes as an adjunct to necessary surgical exploration. This method has a small learning curve, reduces the required incision and helps prevent pushing the FB deeper during dissection. This technique can be replicated in any part of the body for the removal of foreign bodies.

## Declaration of Competing Interest

No Conflicts of interest

## Sources of funding

No funding

## Ethical approval

It was not a trial or a research – so no ethical committee approval was received

## Consent

Written informed consent was obtained from the patient for publication of this case and accompanying images. A copy of this can be presented on request

## Author contribution

Valliappan Muthukumar: Ideology, written and performed the procedure, David Bodansky: writtern and performed the procedure and Ravindra Badge: Consultant incharge, supervised the procedure and paper.

## Registration of research studies

researchregistry5550

## Guarantor

MR Ravi Badge

Consultant – Orthopaedics

Warrington General Hospital

## Provenance and peer review

Not commissioned, externally peer-reviewed

## References

[1] Anderson M.A., Newmeyer W.L., Kilgore E.S. (1982). Diagnosis and treatment of retained foreign bodies in the hand. Am. J. Surg..

[2] Humzah D., Moss A.L. (1994). Delayed digital nerve transection as a result of a retained foreign body. J. Accid. Emerg. Med..

[3] Tantray Mehraj D., Rather Asim (2018). Role of ultrasound in detection of radiolucent foreign bodies in extremities. Strat. Trauma Limb Reconstr..

[4] Bradley M. (2012). Image-guided soft-tissue foreign body extraction - success and pitfalls. Clin. Radiol..

[5] Sharma S., Azzopardi T. (2006). A simple surgical technique for removal of radio-opaque foreign objects from the plantar surface of the foot. Ann. R. Coll. Surg. Engl..

[6] Agha R.A., Borrelli M.R., Farwana R., Koshy K., Fowler A., Orgill D.P. (2018). For the SCARE group. The SCARE 2018 statement: updating consensus surgical CAse REport (SCARE) guidelines. Int. J. Surg..

